# Determinants of low breastfeeding self-efficacy amongst mothers of children aged less than six months: results from the BADUTA study in East Java, Indonesia

**DOI:** 10.1186/s13006-021-00357-5

**Published:** 2021-01-19

**Authors:** Christiana Rialine Titaley, Michael J. Dibley, Iwan Ariawan, Anifatun Mu’asyaroh, Ashraful Alam, Rita Damayanti, Tran Thanh Do, Elaine Ferguson, Kyaw Htet, Mu Li, Aang Sutrisna, Umi Fahmida

**Affiliations:** 1 Faculty of Medicine, Pattimura University, Jl. Ir. M. Putuhena, Poka, Ambon, 97233 Indonesia; 2 Sydney School of Public Health, The University of Sydney, Camperdown, Australia; 3 Center for Health Research, Faculty of Public Health, Universitas Indonesia, Depok, Indonesia; 4 National Institute of Nutrition, Hanoi, 116110 Vietnam; 5 London School of Hygiene and Tropical Medicine, London, WC1E 7HT UK; 6 Southeast Asian Ministers of Education Organization, Regional Center for Food and Nutrition, Pusat Kajian Gizi Regional Universitas Indonesia, Salemba Raya 6, Jakarta, 10430 Indonesia; 7 Global Alliance for Improved Nutrition (GAIN), Jakarta, Indonesia

**Keywords:** Breastfeeding, Self-efficacy, Children aged < 6 months, BADUTA study, Malang District, Sidoarjo District, Indonesia

## Abstract

**Background:**

Despite the increasing rate of exclusive breastfeeding in Indonesia, there is still a need for supportive interventions. The breastfeeding self-efficacy of mothers is a key factor positively associated with optimum breastfeeding practices. Our analysis aims to assess the determinants of low breastfeeding self-efficacy amongst a sample of women with children aged under 6 months in Malang and Sidoarjo Districts, East Java, Indonesia.

**Methods:**

We used information from 1210 mothers of children aged < 6 months recruited in the BADUTA study conducted in 2015–2016 in Malang and Sidoarjo Districts. The outcome variable in this analysis was mothers’ self-efficacy for breastfeeding using the 14 statements in the Breastfeeding Self-Efficacy-Short Form. We evaluated 17 potential predictors of breastfeeding self-efficacy, organized into six sub-groups of variables: (1) context/demographic; (2) household factors; (3) maternal characteristics; (4) child characteristics; (5) breastfeeding practices; and (6) antenatal and delivery care. Logistic regression analyses were employed to examine factors associated with mothers’ self-efficacy with breastfeeding.

**Results:**

More than half of the women in this study had a low level of self-efficacy. One of the factors associated with low breastfeeding self-efficacy found in this study was mothers’ problems related to breastfeeding. Mothers who had problems with breastfeeding not related to illness (adjusted odds ratio [aOR] 3.27; 95% CI 2.45, 4.36) or problems related to both illness and non-illness conditions (aOR 3.57; 95% CI 1.37, 9.33) had higher odds of low breastfeeding self-efficacy than those who did not have any problems. Compared to mothers who completed university education, there was a significantly higher odds of low breastfeeding self-efficacy in mothers who completed primary school or lower (aOR 1.88; 95% CI 1.16, 3.05); completed junior high school (aOR 2.27; 95% CI 1.42, 3.63); and completed senior high school (aOR 1.94; 95% CI 1.29, 2.91). Other significant predictors of low breastfeeding self-efficacy were mothers not exposed to any breastfeeding interventions (aOR 1.87; 95% CI 1.09, 3.22); working outside the house (aOR 1.69; 95% CI 1.23, 2.32); not obtaining any advice on breastfeeding (aOR 1.40; 95% CI 1.08, 1.82); with low knowledge of breastfeeding (aOR 1.38; 95% CI 1.03, 1.84); and delivered by Caesarean section (aOR 1.34; 95% CI 1.05, 1.70).

**Conclusions:**

Multipronged breastfeeding education programs and support are required to improve women’s self-efficacy with breastfeeding. Improved access to breastfeeding counselors, active support for mothers following cesarean delivery, and increased supporting facilities at workplaces are essential to improve self-efficacy with breastfeeding.

**Supplementary Information:**

The online version contains supplementary material available at 10.1186/s13006-021-00357-5.

## Background

The importance of breastfeeding for both babies and mothers has been widely acknowledged [1]. For infants, exclusive breastfeeding is strongly recommended in their first 6 months of life to provide the ideal nutrition for optimal growth and development [2, 3]. After this period, infants should continue to receive breast milk and appropriate complementary feeding until reaching at least 2 years of age [4]. The short-term and long-term effects of breastfeeding for babies include the short-term effects of reductions in the risk of diarrhea and respiratory infections [5], the long-term effects of protection against overweight and obesity, as well as a positive effect on intelligence [6]. For mothers, breastfeeding helps to increase child spacing, and reduce the risk of mastitis, postpartum hemorrhage, depression, and ovarian and breast cancer [4, 7, 8].

In Indonesia, the rate of exclusive breastfeeding is increasing, and contributing to this change is the Decree of the Minister of Health of the Republic of Indonesia No.450/MENKES/SK/IV/2004 concerning exclusive breastfeeding for infants, which contains the ten steps towards successful breastfeeding [9]. Subsequently, the Indonesian Government issued Regulation No. 33 Year 2012, declaring that exclusive breastfeeding fulfilled the baby’s right to get the best food source until the age of 6 months [10]. However, despite the improvement in breastfeeding practices, supportive interventions are still required. Based on the last two Indonesia Demographic and Health Survey data, the national rate of exclusive breastfeeding has increased from 32.4% in 2007 [11] to 41.5% in 2012 [12] and to 52.0% in 2017 [12]. However, the 2018 Basic Health Survey from the Ministry of Health reported the national rate of exclusive breastfeeding amongst infants aged 0–5 months was 74.5%, ranging from 72.7% in urban areas to 76.6% in rural areas [13].

There is a range of different factors associated with breastfeeding practices, including the intention to breastfeed, maternal age, maternal education, smoking status, economic status, knowledge of breastfeeding, advice from health professionals, problems encountered with breastfeeding, or child’s birthweight [14]. One of the factors also reported positively associated with breastfeeding is the mothers’ breastfeeding self-efficacy. Self-efficacy, an element of the social cognitive theory of Bandura [15], is a predictor of health-related behaviors [16]. Self-efficacy consists of two components: (1) the outcome expectancy or the belief that a given behavior will produce a particular outcome, and (2) self-efficacy expectancy or an individual’s conviction that they can successfully perform certain tasks or behaviors to produce the desired outcome [15, 16]. We defined breastfeeding self-efficacy as the mothers’ beliefs and confidence in their ability to successfully breastfeed their infants [17]. Breastfeeding self-efficacy is an important predictor of the duration [18] and the exclusivity of breastfeeding [19, 20]. Thus, mothers’ breastfeeding self-efficacy assessment will help identify those women who need more support for breastfeeding during the postnatal period [18].

The Global Alliance for Improved Nutrition (GAIN) and the University of Sydney, in collaboration with the Centre for Health Research, Universitas Indonesia (CHR-UI), SEAMEO-RECFON, and the London School Hygiene and Tropical Medicine, conducted the BADUTA Study, an impact evaluation of the BADUTA Program, in 2015 to 2017 in East Java, Indonesia [21]. Our analysis used the cross-sectional surveys conducted for the BADUTA Program evaluation. It aimed to examine the factors associated with low breastfeeding self-efficacy amongst the sample of women with children aged under 6 months in Malang and Sidoarjo Districts, East Java, Indonesia.

## Methods

### Data source and study sites

This analysis used data derived from the BADUTA study conducted in 2015–2016 in Sidoarjo and Malang District of East Java, Indonesia. The Ministry of Health, Republic of Indonesia identified Malang and Sidoarjo Districts as the study sites for evaluating the BADUTA program since they represented peri-urban and rural areas of East Java Province. In both districts, we selected six sub-districts to conduct the trial. The sub-districts in Sidoarjo District were Tulangan, Wonoayu, Sidoarjo, Prambon, Taman, and Krian; and in Malang District were Dampit, Turen, Tumpang, Poncokusumo, Gondanglegi, and Jabang.

We have presented detailed information about the BADUTA study protocol elsewhere [21]. We used data for this analysis from two independent cross-sectional surveys conducted in 2015 at the beginning and 2017 at the end of the project. To assess breastfeeding self-efficacy amongst mothers, we only used information collected from mothers of children less than 6 months of age.

### Background information on study sites

East Java Province is one of the provinces in Indonesia located on Java Island, and the capital is Surabaya City, the second-largest city in Indonesia. East Java’s total population is approximately 37 million people, the second-most populous province in the country [22]. Malang District, located in the center-south region of East Java Province, has an estimated total population in 2017 of 2,576,596 people [23]. Most of the people were working as laborers or private employees (37.63%) [23]. Sidoarjo District, located north of Surabaya City, has an estimated total population in 2017 of 2,207,600 people [24].

### Study design and samples of the study

We conducted an observational epidemiological study to examine factors associated with low breastfeeding self-efficacy. We combined the data from the baseline and endline cross-sectional surveys for both the intervention and comparison groups in the BADUTA study for our analysis.

The sampling design in this trial used a three-stage cluster sampling procedure. In each of the twelve subdistricts selected at the initial stage, we selected ten villages using the probability proportionate to size sampling method. Next, we selected two sub-villages from each chosen village using simple random sampling method. Finally, we conducted a mini census to list all children aged < 2 years, in each of the selected sub-villages. Using the listing as a sampling frame, we selected 12 children aged < 2 years and their mothers using simple random sampling.

In the baseline survey of the BADUTA study, the sample size for children under 2 years old was 2435 children, while in the endline survey, the sample for children under 2 years old was 2740 [21]. We only used information from 1210 women with children under 6 months (575 from the baseline and 635 from the endline survey) for this analysis.

### Survey instruments and field personnel

The field team carried out house-to-house interviews using pretested and structured questionnaires. The information collected in this study included socio-economic and demographic characteristics; infant feeding practices as well as the intention of the mother to breastfeed and self-efficacy for breastfeeding of the mothers; child morbidity, reported by mother/caregiver; as well as contact with the health system and exposure to the interventions. Information about the mothers’ self-efficacy for breastfeeding was collected using the Breastfeeding Self-Efficacy Scale-Short Form questionnaire developed by Dennis [16], a 14-item instrument to measure breastfeeding confidence.

At the baseline, we established eight fieldwork teams in each district. However, in the endline study, we established ten fieldwork teams to shorten data collection duration. Each team consisted of one field coordinator, one assistant field coordinator, and ten enumerators for interviews. There were 10 field coordinators, 130 interviewers, and 20 nurses or midwives recruited [21]. The nurses and midwives collected the blood samples and took anthropometric measurements.

Before data collection, all field workers attended a one-week training program to standardize the enumerators, and their coordinators, with the questionnaire, sampling methodology, and interview techniques. The training covered different aspects of the study, i.e., an overview of the BADUTA study, the use of CommCare application, household listing and data collection procedures, explanations of study instruments (listing forms and questionnaires), quality controls for data collection, as well as a field plan. The training program included a two-day tryout to allow all training participants to practice the household listing and interviews using the CommCare application. A pre and post-test were also carried out before and after the training sessions, respectively. Enumerators with low post-test results were monitored closely by field coordinators and supervisors, particularly at the beginning of data collection, to ensure their ability and quality to conduct all fieldwork activities.

Data were collected electronically on hand-held devices using the CommCare system developed by Dimagi [25]. Information was recorded on structured, error detecting forms on tablets and then dispatched directly to a server to clean and merge. Field supervisors and a data manager monitored the data quality regularly.

### Outcome variable

This analysis’s outcome variable was mothers’ self-efficacy for breastfeeding as a binary variable (low or high self-efficacy on breastfeeding). We defined breastfeeding self-efficacy as the mothers’ beliefs and confidence in their ability to breastfeed their infants successfully. Information about the mothers’ self-efficacy for breastfeeding was collected using the Breastfeeding Self-Efficacy Scale-Short Form [16]. For each of the 14 statements, we asked the mothers to give a score from 1 to 5 that offered a range of answer options from “strongly disagree” to “strongly agree,” respectively. We added all the scores to calculate the total score. As in other studies, we based the breastfeeding self-efficacy classification on the median of the total score [26, 27]. Previous studies supported using either the mean or the median as the cut-off point to categorize low and high breastfeeding self-efficacy [27–29]. We classified mothers scoring less than the median as having a low self-efficacy on breastfeeding. Those scoring equal to or above the median we classified as having a high self-efficacy.

### Potential predictors

**Fig. 1:**
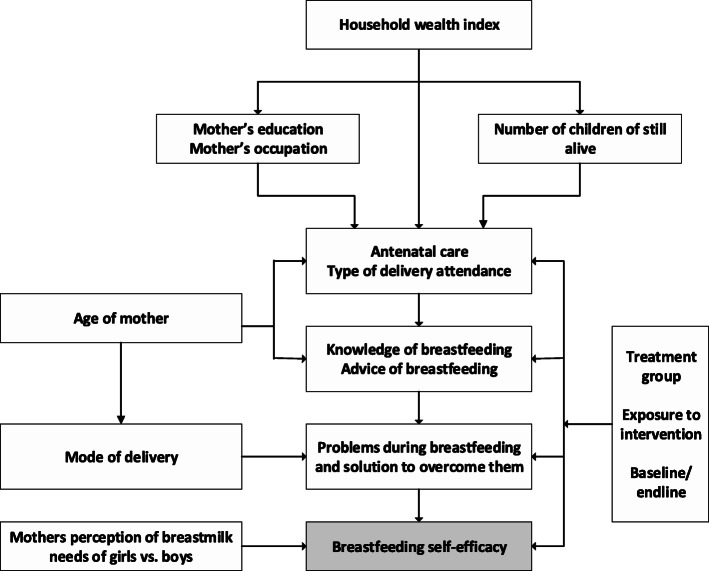
The analytical framework used to examine factors associated with mothers’ low self-efficacy on breastfeeding, The BADUTA Study in East Java, Indonesia, 2015–2016

The potential predictors were selected using the analytical framework shown in Fig. 1. In total, there were 17 potential predictors of breastfeeding self-efficacy included in the analyses, categorized into six sub-groups: (1) context/demographic variables; (2) household characteristics; (3) maternal characteristics; (4) child characteristics; (5) breastfeeding characteristics; and (6) antenatal and delivery care characteristics.

In the group of contextual and intervention variables, we constructed a composite variable indicating the total number of interventions from 13 variables representing breastfeeding-related interventions in the BADUTA study. Those 13 interventions were: (1) discussing breastfeeding with cadres on a home visit during pregnancy; (2) discussing exclusive breastfeeding in pregnant women’s class during pregnancy; (3) did not receive any free formula milk after delivery (part of the Baby Friendly Hospital Initiative); (4) discussing breastfeeding with a village facilitator during pregnancy; (5) watching a breastfeeding-related video shown by the village facilitator during pregnancy; (6) discussing the topic of breastfeeding in emo-demo sessions; (7) receiving mobile phone messages on early initiation of breastfeeding; (8) receiving mobile phone messages on the benefits of colostrum; (9) receiving mobile phone messages on exclusive breastfeeding; (10) receiving mobile phone messages on exclusive breastfeeding problems and how to handle them; (11) receive breastfeeding counseling by midwives during pregnancy; (12) receive breastfeeding counseling by cadres during pregnancy; and (13) watching TV commercials about breastfeeding. For each question, we scored the answers one if the mothers answered “yes,” and scored zero if answered otherwise. We then summed all the scores to obtain a total intervention score. We then categorized the total intervention score for each individual into “no intervention” (total score = 0); “one intervention” (total score = 1); “two interventions” (total score = 2), and “three or more intervention” (total score is ≥3). Finally, we calculated the total intervention score for all women from both the intervention and comparison groups included in this analysis. Our purpose was to assess any breastfeeding intervention’s impact, whether from the study interventions or routine programs, on breastfeeding self-efficacy. We have documented a detailed explanation of the interventions in the BADUTA study elsewhere [21].

In household characteristics, we constructed the household wealth index variable using Principal Component Analysis (PCA) [30] from an inventory of the household’s facilities and assets. These items included ownership of electricity, drinking water, toilet facility, type of toilet facility, fecal final disposal, and ownership of bicycle, television, water heater, 12 kg of LPG, fridge, and car. We ranked households by this index and classified them into five quintiles, i.e., poorest, poor, middle, rich, and richest categories of households.

In the breastfeeding knowledge and experience group, we developed one composite variable to represent mothers’ knowledge about breastfeeding. We constructed this variable from five questions: (1) the best food or liquid to be provided to children aged < 6 months; (2) the duration for exclusively breastfeeding a child; (3) the duration a child should receive breast milk; (4) the benefits of giving breast milk to children; and (5) the time a child should receive complementary feeding. A score of one was assigned to all correct answers and zero for all incorrect answers for each question. We summed all the scores to get the total knowledge score, and we calculated the median value. We developed two categories of knowledge: (1) a high level of knowledge for those whose total knowledge score was greater or equal to the median, and (2) a low level of knowledge for those whose total knowledge score was less than the median. To test if previous experience with feeding infants influenced breastfeeding self-efficacy, we also used an indicator for previous live births as we did not specifically ask the mothers about their earlier breastfeeding experiences. For the variable of problems with breastfeeding, we categorized mothers into four groups: (1) Mothers who did not experience any problems with breastfeeding; (2) Mothers who had breastfeeding problems not related to illness; (3) Mothers who had breastfeeding problems related to illness or anatomical conditions; and (4) Mothers who had both types of problems. We categorized mothers as having as “breastfeeding problems not related to illness, who mentioned their breastmilk was insufficient, or they could not express it, or the infant refused breastfeeds. We categorized mothers with problems due to swollen breasts/mastitis, sore nipples, or flat/embedded/large nipples as a “problem related to illness/anatomical conditions.” We categorized mothers reporting both types of breastfeeding problems as “mothers who had both types of problems.

### Data analysis

To examine the characteristics of all variables (outcome variables and potential predictors) used in the analysis, we used contingency tables. We then applied logistic regression analyses to determine factors associated with all outcome variables using odds ratios (ORs) as the estimated measure of association. We used Stata survey commands (svyset) to adjust for the clustering from the cluster randomization. All estimates presented in this analysis considered the complex sample design.

In the first step of logistic regression, we used bivariate analyses to independently assess the relationship between outcome variables and their potential predictors. In the second step, we performed multivariate analyses using a backward elimination method to remove all variables not significantly related to the study outcome, with a significance level of 0.05. Two variables selected a priori and retained in the final model regardless of the significance level were: (1) Period of the survey (baseline or endline) and (2) the fulfillment of the minimum requirement of four antenatal care visits by trimester (met or did not met). We obtained the adjusted ORs (aOR) and 95% confidence intervals [CI] (95% CIs) for all the final model variables.

In multivariate analysis, we used problems of breastfeeding and the number of breastfeeding interventions as composite variables. After obtaining the final model (Model #1), we developed the second model by replacing breastfeeding problems with each type of breastfeeding problem (Model #2). We also developed the third model by replacing the breastfeeding intervention variable with all the individual exposure to intervention indicators (Model #3). We then retained the other variables in the final model of Model #1 in Model #2 and Model #3. We used Stata/MP software (version 13.1; Stata Corp) for all analyses.

### Collaborating institutions

This study was conducted by an International Research Consortium that comprised of experienced researchers from the University of Sydney (Australia), the London School of Hygiene and Tropical Medicine (LSHTM) (United Kingdom), the Center for Health Research Universitas Indonesia (CHR-UI), the Indonesia Nutrition Foundation for Food Fortification (KFI), and the Southeast Asian Ministers of Education Organization (SEAMEO), Regional Center for Food and Nutrition (RECFON).

## Results

**Fig. 2:**
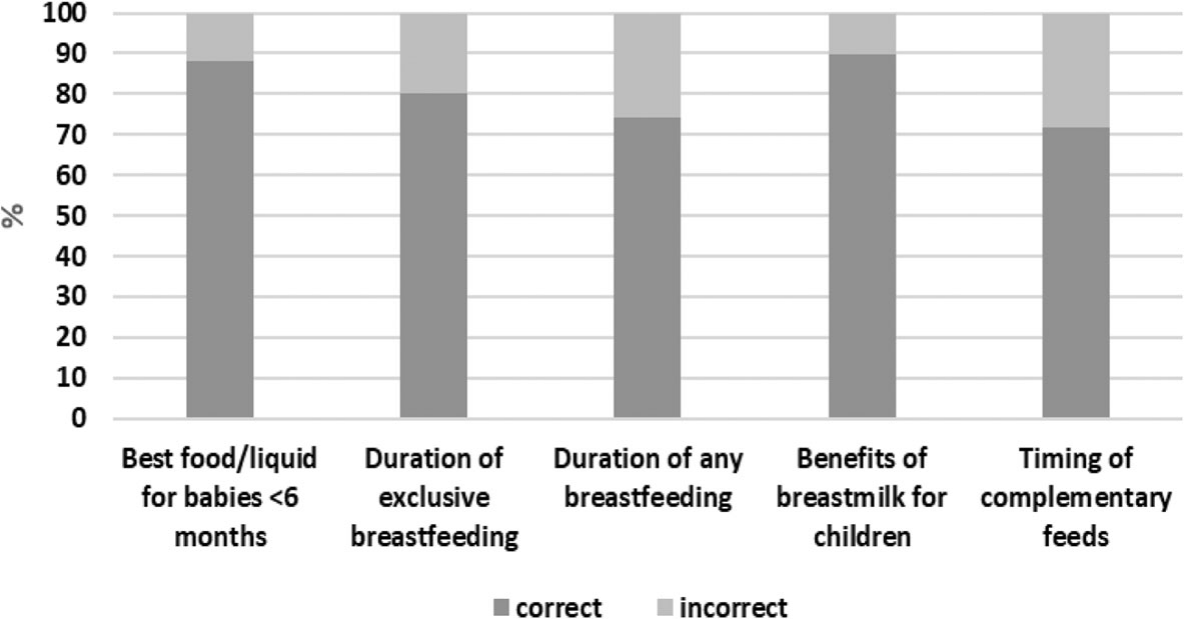
Distribution of mothers of children under six months by their answers to each breastfeeding knowledge component, The BADUTA Study in East Java, Indonesia, 2015–2016

Table 1 shows the frequency distribution of mothers with children under 6 months by households, mothers, child characteristics, breastfeeding experience and knowledge, antenatal and delivery care services, and women with low breastfeeding self-efficacy. Approximately three-quarters of the mothers were aged 20–34 years, and the majority were housewives. Slightly more than half reported ever receiving some advice on breastfeeding. Almost 31% of mothers had problems with breastfeeding: 16.9% of mothers had problems not related to illness; 11.3% had problems related to illness or anatomical condition, and 2.5% had both types of problems. Around 25% of mothers had a low level of knowledge regarding breastfeeding. The characteristics of women with low breastfeeding self-efficacy were similar to all the women in the study except more worked outside their house, more had low levels of knowledge about breastfeeding, and more had experienced problems with breastfeeding. From the six knowledge components used in this analysis, the lowest percentage of correct answers was regarding the time to start complementary feeding (71.7%), followed by the minimum duration children should receive breast milk (74.3%) (Fig. 2).

**Table 1:** Frequency distribution of mothers with children under six months old by households, mothers, child characteristics, breastfeeding experience, and knowledge, antenatal and delivery care services as well as breastfeeding self-efficacy status, The BADUTA Study in East Java, Indonesia, 2015–2016

Variables	Total Sample		Breastfeeding Self-Efficacy^a^		
	***n***	%	Low (%)	High (%)	***p***
**Household characteristics**					
**Household wealth index**					
Poorest	243	20.1	54.7	45.3	*0.086*
Poor	281	23.2	54.5	45.5	
Middle	236	19.5	59.8	40.2	
Rich	268	22.2	61.9	38.1	
Richest	182	15.0	50.0	50.0	
**Maternal characteristics**					
**Maternal age**					
≤ 19 years	85	7.0	64.7	35.3	*0.280*
20–34 years	913	75.5	56.1	43.3	
35+ years	212	17.5	55.2	44.8	
**Maternal education**					
University/Academy	154	12.7	44.2	55.8	*0.008*
Completed senior high school	495	40.9	57.2	42.8	
Completed junior high school	334	27.6	60.5	39.5	
No school/incomplete primary/completed primary school	227	18.8	57.7	42.3	
**Maternal occupation**					
Housewife	963	79.6	54.9	45.1	*0.027*
Working outside the house	247	20.4	62.8	37.2	
**Number of children still alive**					
1	534	44.1	56.6	43.4	*0.993*
2	477	39.4	56.2	43.8	
3	156	12.9	57.1	42.9	
4+	43	3.6	58.1	41.9	
**Previous live birth**					
None	520	43.0	56.4	43.6	*0.911*
Any	690	57.0	56.7	43.3	
**Antenatal, delivery care**					
**Minimum antenatal care visits**^b^					
Completed (4+ visits)	858	71.6	54.6	45.4	*0.057*
Incomplete (< 4 visits)	340	28.4	60.6	39.4	
**Mode of delivery**					
Normal	821	67.9	55.2	44.8	*0.168*
Caesarea	389	32.2	59.4	40.6	
**Birth attendant**					
General Practitioner/OBGYN	483	39.9	57.1	42.3	*0.772*
Midwife/nurse	708	58.5	55.9	44.1	
Traditional birth attendant/family/friend	19	1.6	63.2	36.8	
**Child’s characteristics**					
**Sex of the child**					
Male	582	48.7	56.0	44.0	*0.792*
Female	613	51.3	56.8	43.2	
**Birthweight according to monitoring card**					
Larger than average	275	22.8	62.6	37.4	*0.062*
Average	831	69.0	54.5	45.5	
Smaller than average	99	8.2	54.6	45.4	
**Breastfeeding experience and knowledge**					
**Ever received any advice on breastfeeding**					
Yes	638	52.7	52.7	47.3	*0.004*
No	572	47.3	60.8	39.2	
**Knowledge about breastfeeding**					
High^c^	909	75.1	53.8	46.2	*0.001*
Low^d^	301	24.9	64.8	35.2	
**Problems with breastfeeding**					
None	838	69.3	50.8	49.2	*< 0.001*
Not related to illness	204	16.9	76.5	23.5	
Related to illness/anatomical condition	137	11.3	56.9	43.1	
Both types of problems	30	2.5	76.7	23.3	

Note:^a^Low breastfeeding self-efficacy was mothers whose total self-efficacy score was less than the median value. ^b^Minimum Antenatal Care refers to the recommendation of at least four antenatal visits, i.e., once in trimester one to three, and twice in trimester three. ^c^High level of knowledge was mothers whose total knowledge score was greater than, or equal to the median knowledge score value. ^d^Low level of knowledge was mothers whose total knowledge score was less than the median knowledge score value

Table 2 presents the distribution of 1210 (575 from the baseline and 635 from the endline surveys) mothers of children aged less than 6 months who were interviewed in the BADUTA study by contextual, intervention, and breastfeeding characteristics against low self-efficacy status. Based on the interventions received, 40% received at least one breastfeeding intervention, but less than 8% received three or more interventions. The low percentage of mothers receiving BADUTA interventions is due to the pooled dataset from baseline and endline surveys. At the endline survey, around 33% of women in the comparison group did not receive any breastfeeding interventions, while 67% received at least one type of intervention (47% received one; 17% received two, and 4% received three or more). In the intervention group, 13% of women did not receive any breastfeeding interventions, and 87% received at least one intervention (36% received one; 24% received two, and 27% received three or more interventions).

**Table 2:** Frequency distribution of mothers with children under six months by contextual and intervention characteristics and breastfeeding and self-efficacy status, The BADUTA Study in East Java, Indonesia, 2015–2016

Variable	Total Sample		Breastfeeding Self-Efficacy^a^		
	***n***	%	Low (%)	High (%)	***p***
**Exposure to interventions**					
Exposed to intervention^b^	286	23.6	49.7	50.3	*0.007*
Not exposed to intervention^c^	924	76.4	58.7	41.3	
**Number of breastfeeding interventions mothers were exposed to**					
Three or more interventions	91	7.5	38.5	61.5	*< 0.001*
Two interventions	127	10.5	48.8	51.2	
One intervention	266	22.0	56.4	43.6	
No interventions	726	60.0	60.2	39.8	
**Period**					
Baseline	575	47.5	60.5	39.5	*0.008*
Endline	635	52.5	52.9	47.1	
**Ever visited by village facilitator at home**					
Yes	22	1.8	36.4	63.6	*0.054*
**Ever attended “emo-demo.”**					
Yes	50	4.1	44.0	56.0	*0.068*
**Ever received SMS Bunda messages**					
Yes	20	1.7	35.0	65.0	*0.050*
**Ever visited by cadre at home**					
Yes	17	1.4	29.4	70.6	*0.023*
**Ever attended pregnancy class**					
Yes	40	3.3	40.0	60.0	*0.032*
**Roomed in with baby after delivery**					
Yes	363	30.0	48.2	51.8	*< 0.001*
**Ever received counselling by a midwife**					
Yes	188	15.5	52.1	47.9	*0.185*
**Ever received counselling by a cadre**					
Yes	24	2.0	37.5	62.5	*0.058*
**Ever seen “*Rumpi Sehat*” TV commercials**					
Yes	123	10.2	43.9	56.1	*0.003*

Note:^a^Low self-efficacy was mothers whose total self-efficacy score was less than the median value. ^b^Exposed to intervention refers to respondents living in the intervention sub-districts at the endline survey. ^c^Not exposed to intervention referred to all respondents from the baseline survey and those living in the control sub-districts at the endline survey

**Fig. 3:**
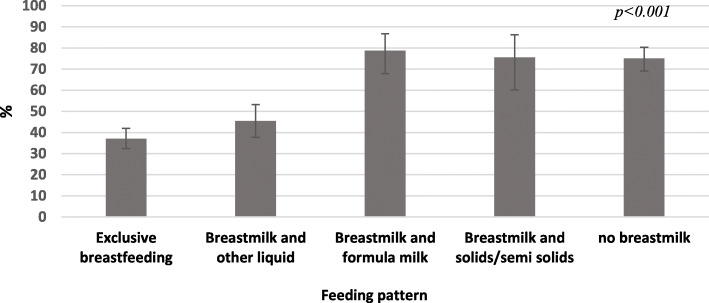
Distribution of mothers with low breastfeeding self-efficacy by the feeding patterns of their infants under six months of age, The BADUTA Study in East Java, Indonesia, 2015–2016. Note: ^1)^Based on mothers’ 24-h recall; ^2)^Without formula milk/solids/semi-solids; ^3)^Can include liquid other than formula milk, yet not solids/semi-solids; ^4)^Can include formula milk/other liquid. We used the chi-square statistic to examine the distribution of infants who were exclusively breastfed based on mothers’ self-efficacy level. The bars represent 95% CI. We used STATA survey commands (svy) to adjust for the cluster sampling design

Over half the women (56%) in the study had low breastfeeding self-efficacy. The median score of breastfeeding self-efficacy in mothers with low efficacy was 35 (Standard Deviation [SD] = 6.33) and in mothers with high self-efficacy was 43 (SD = 4.31). As shown in Fig. 3, there was a significant difference in the percentage of women with low breastfeeding self-efficacy related to how they were feeding their infants under 6 months of age (*p* < 0.001). There was no significant difference between the percentage of mothers with low breastfeeding self-efficacy who exclusively or predominantly breastfed their infants. However, the percentage of mothers with low breastfeeding self-efficacy was significantly higher among women who were exclusively or predominantly breastfeeding compared with women feeding with breast milk and formula, or with breast milk and solids/semi-solids, or not breastfeeding at all (Fig. 3).

**Fig. 4:**
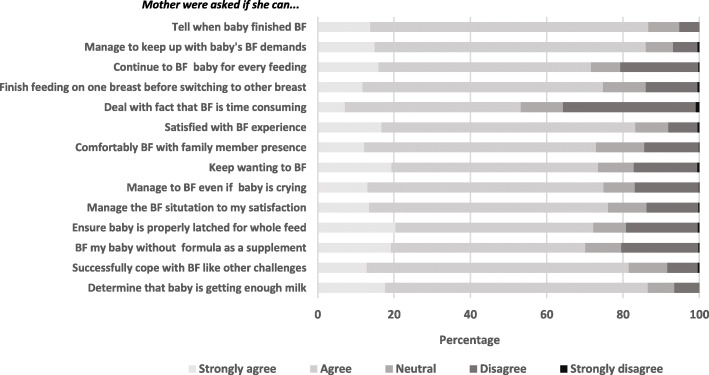
Distribution of mothers of children under six months by their answers to each component of breastfeeding self-efficacy short form (BSES-SF), The BADUTA Study in East Java, Indonesia, 2015–2016. Note: For each question, a score of 1 to 5 was assigned to the responses from “strongly disagree” to “strongly agree.” BF = breastfeeding

Figure 4 presents the distribution of the respondents’ answers to each component of the Breastfeeding Self-Efficacy Scale. More than 50% of the mothers answered either agree or strongly agree with all components of the Breastfeeding Self-Efficacy Scale score. We found the highest percentage of agreement in mothers who felt able to tell when their baby finished breastfeeding (80%), and the lowest was in mothers who felt they could deal with breastfeeding being time-consuming. As shown in Table 1, low breastfeeding self-efficacy was highest among women with lower education levels, of younger age, and working outside the house. Low breastfeeding efficacy declined as the number of breastfeeding interventions the women received increased.

Table 3 presents the results of the analysis of factors associated with low breastfeeding self-efficacy. One of the factors that were significantly related to low mothers’ breastfeeding self-efficacy was problems encountered related to breastfeeding. There was no association between mothers who had breastfeeding problems related to illness or anatomical conditions and low breastfeeding self-efficacy (aOR 1.36; 95% CI 0.95, 1.93, *p* = 0.089). However, there was a significant association between mothers who had problems not related to illness (aOR 3.27; 95% CI 2.45, 4.36, *p* < 0.001), or both types of problems (aOR 3.57; 95% CI 1.37, 9.33, *p* = 0.010) and low breastfeeding self-efficacy. When the ‘problems with breastfeeding’ variable was replaced in the final model by each type of mothers’ breastfeeding problem, we found an increased odds for low breastfeeding self-efficacy for mothers with a swollen breast (aOR 3.10; 95% CI 1.33, 7.21, *p* = 0.009); with flat nipples (aOR 2.75; 95% CI 1.29, 5.87, *p* = 0.009); whose infants refused breastfeeds (aOR 5.45; 95% CI 1.64, 18.13, *p* = 0.006); and who perceived their breastmilk is not enough (aOR 2.80; 95% CI 2.04, 3.84, *p* < 0.001) (Supplementary Table 1).

**Table 3:** Factors associated with low breastfeeding self-efficacy amongst mothers of children under six months old, The BADUTA Study in East Java, Indonesia, 2015–2016

Variable	Univariate				Multivariate^a^			
	aOR	95% CI		***p***	aOR	95% CI		***p***
**Contextual and intervention characteristics**								
**Exposure to intervention**								
Exposed to intervention^b^	1.00							
Not exposed to intervention^c^	1.44	1.13	1.83	*0.003*				
**Number of breastfeeding interventions mothers exposed to**								
Three or more interventions	1.00				1.00			
Two interventions	1.53	0.83,	2.81	*0.175*	1.47	0.74,	2.89	*0.269*
One intervention	2.07	1.16,	3.69	*0.014*	1.81	0.98,	3.35	*0.059*
No interventions	2.42	1.41,	4.15	*0.001*	1.87	1.09,	3.22	*0.024*
**Period**								
Baseline	1.00				1.00			
Endline	0.73	0.55,	0.97	*0.032*	0.89	0.61,	1.28	*0.517*
**Household characteristics**								
**Household wealth index**								
Poorest	1.00							
Poor	0.99	0.70,	1.39	*0.948*				
Middle	1.23	0.86,	1.75	*0.257*				
Rich	1.35	0.93,	1.95	*0.118*				
Richest	0.83	0.52,	1.32	*0.426*				
**Mother’s characteristics**								
**Maternal age**								
≤ 19 years	1.00							
20–34 years	0.70	0.41,	1.17	*0.172*				
35+ years	0.67	0.40,	1.14	*0.138*				
**Maternal education**								
University/Academy	1.00				1.00			
Completed senior high school	1.69	1.19,	2.39	*0.003*	1.94	1.29,	2.91	*0.002*
Completed junior high school	1.94	1.31,	2.86	*0.001*	2.27	1.42,	3.63	*0.001*
No school/incomplete primary/completed primary school	1.73	1.12,	2.67	*0.014*	1.88	1.16,	3.05	*0.011*
**Maternal occupation**								
Housework	1.00				1.00			
Working outside the house	1.38	1.06,	1.81	*0.018*	1.69	1.23,	2.32	*0.001*
**Number of children still alive**								
1	1.00							
2	0.99	0.77,	1.26	*0.904*				
3	1.02	0.69,	1.52	*0.920*				
4+	1.07	0.55,	2.07	*0.847*				
**Previous live birth**								
None	1.00							
Any	1.01	0.82,	1.26	*0.906*				
**Antenatal and delivery care**								
**Minimum antenatal care visits**^d^								
Completed (4+ visits)	1.00				1.00			
Incomplete (< 4 visits)	1.28	0.98,	1.68	*0.073*	1.23	0.92,	1.65	*0.166*
**Mode of delivery**								
Normal	1.00				1.00			
Caesarean	1.19	0.96,	1.47	*0.108*	1.34	1.05,	1.70	*0.017*
**Birth attendant**								
General practitioner/OBGYN	1.00							
Midwife/nurse	0.95	0.78,	1.165	*0.631*				
Traditional birth attendant/family/friend	1.29	0.54,	3.082	*0.572*				
**Child’s characteristics**								
**Sex of the child**								
Male	1.00							
Female	1.03	0.82,	1.30	*0.791*				
**Birthweight from monitoring card**								
Larger than average	1.00							
Average	0.72	0.51,	1.00	*0.053*				
Smaller than average	0.72	0.44,	1.17	*0.183*				
**Breastfeeding knowledge & experience**								
**Ever received any breastfeeding advice**								
Yes	1.00				1.00			
No	1.40	1.09,	1.78	*0.007*	1.40	1.08,	1.82	*0.013*
**Knowledge about breastfeeding**								
High level^e^	1.00				1.00			
Low level^f^	1.58	1.19,	2.10	*0.002*	1.38	1.03,	1.84	*0.029*
**Problems with breastfeeding**								
None	1.00				1.00			
Not related to illness	3.14	2.34,	4.23	*< 0.001*	3.27	2.45,	4.36	*< 0.001*
Related to illness/anatomical condition	1.28	0.91,	1.80	*0.158*	1.36	0.95,	1.93	*0.089*
Both types of problems	3.18	1.29,	7.81	*0.012*	3.57	1.37,	9.33	*0.010*

Note:^a^Multivariate logistic regression using the backward elimination method to select significant predictors of low breastfeeding self-efficacy. The variable for the minimum requirement of four antenatal care visits by trimester was selected a priori to be retained in the final model regardless of its significance level. ^b^Exposed to intervention refers to respondents living in the intervention sub-districts at the endline survey ^c^Not exposed to intervention referred to all respondents from the baseline survey and those living in the control sub-districts at the endline survey. ^d^Minimum antenatal care refers to the recommendation of at least four antenatal visits, i.e., once in trimester one to three, and twice in trimester three. ^e^High level of knowledge was mothers whose total knowledge score was greater than, or equal to the median knowledge score value. ^f^Low level of knowledge was mothers whose total knowledge score was less than the median knowledge score value

The level of mothers’ education was also significantly associated with low breastfeeding self-efficacy. A higher odds was found in mothers who completed primary school or lower (aOR 1.88; 95% CI 1.16, 3.05, *p* = 0.011); completed junior high school (aOR 2.27; 95% CI 1.42, 3.63, *p* = 0.001), and completed senior high school (aOR 1.94; 95% CI 1.29, 2.91, *p* = 0.002), compared to mothers who completed university education.

As expected, the likelihood of having low breastfeeding self-efficacy reduced as the mothers’ number of breastfeeding interventions increased. The odds of low breastfeeding self-efficacy significantly increased in mothers not exposed to any breastfeeding interventions (aOR 1.87; 95% CI 1.09, 3.22, *p* = 0.024). In the analysis shown in the Supplementary Table 2, we replaced the total number of interventions with an indicator of exposure for each specific intervention. It is interesting to see that in general, there was no significant association between each intervention and self-efficacy, except for mothers visited by a cadre at home to talk about breastfeeding (aOR 0.28; 95% CI 0.09, 0.85, *p* = 0.025).

Moreover, we found an increased odds of low breastfeeding self-efficacy in mothers working outside the house (aOR 1.69; 95% CI 1.23, 2.32, *p* = 0.001); who had never received any breastfeeding advice (aOR 1.40; 95% CI 1.08, 1.82, *p* = 0.013); and who had low knowledge of breastfeeding (aOR 1.38; 95% CI 1.03, 1.84, *p* = 0.029). The odds of low breastfeeding self-efficacy were also higher in mothers delivered by a Caesarean section (aOR 1.34; 95% CI 1.05, 1.70, *p* = 0.017) than those with normal delivery (Table 3).

## Discussion

### Main findings

Our study found that more than half of the women with children under 6 months had a low breastfeeding self-efficacy level. It was also confirmed by the exclusive breastfeeding status of mothers, as a significantly higher percentage of mothers with high breastfeeding self-efficacy exclusively breastfed their babies than those with low breastfeeding self-efficacy. The significant predictors of low breastfeeding self-efficacy included low education level, working outside the house, never receiving any advice on breastfeeding, low knowledge about breastfeeding, and breastfeeding problems. Mothers who had a cesarean section delivery also had a low self-efficacy for breastfeeding. As expected, the odds of having low self-efficacy were lower as the number of breastfeeding interventions received increased. This study’s findings should assist decision-makers and program managers in designing and implementing supportive interventions to increase mothers’ self-efficacy with breastfeeding. Increased self-efficacy will promote and accelerate the improvement of breastfeeding practices to the benefit of both mothers and their children.

### The role of knowledge and education in breastfeeding self-efficacy

We confirmed the relationship between knowledge and breastfeeding self-efficacy in our study. Firstly, mothers with a low level of breastfeeding knowledge were more likely to have lower self-efficacy than those with a high level of knowledge. Secondly, we found an association between low self-efficacy with lower educational attainments. The level of education mirrors mothers’ level of knowledge and awareness about breastfeeding and health in general, the possibility of having more exposure to health-related information, and comprehension about health information received. A study has reported that mothers with secondary and high school education are more likely to have a higher self-efficacy score than those graduating from university [31]. The difference was assumed due to the mother’s occupation, as highly educated mothers were more likely to have a job and work outside the house. However, in our analysis, we adjusted the association between education or knowledge and breastfeeding self-efficacy for the maternal occupation to remove the confounding of the effects by occupation.

### Education-based interventions and support to improve breastfeeding self-efficacy

The important role of knowledge and education in our study indicates the importance of promoting strategies to enhance mothers’ and other family members’ awareness of breastfeeding. Previous literature has reported the benefits of prenatal [32–35] and postnatal education and the support mothers received for breastfeeding [36, 37]. It is also important to consider the type, timing, setting, and frequency of education interventions [33]. A systematic review highlighted the importance of support-based initiatives during postnatal care through interaction with lactation experts [38]. The use of combined settings (health facility and community) for health education, not solely in hospitals or community, was reported to be more beneficial than education merely in a health facility [33, 39].

Furthermore, we found that mothers who had never received any advice on breastfeeding were more likely to have lower breastfeeding self-efficacy than those who had ever received any advice. Interactive and face-to-face education, in addition to the consistent delivery of breastfeeding messages, will increase self-efficacy. This finding shows the need to improve and strengthen counseling activities on breastfeeding by health workers. Quality counseling programs will help mothers to improve their confidence in breastfeeding. Moreover, previous literature has revealed that combined individual and group counseling is more effective than individual or group counseling only [39]. Multiple intervention contacts, rather than only a single contact intervention, have more favorable outcomes [33, 40]. Thereby, health workers should effectively use every contact opportunity with mothers and other family members, from antenatal to postnatal period, to improve mothers’ awareness and self-efficacy with breastfeeding.

One review found that peer support interventions were also effective in promoting optimum breastfeeding [40], suggesting an opportunity to encourage health-volunteers and mothers, who have successfully breastfed, to support other mothers breastfeeding their children. This analysis found that mothers with breastfeeding problems had a lower self-efficacy than those who never experienced any problems. The problems included those not related to illness, such as mothers whose infants refused to breastfeed, and mothers who perceived their breastmilk was not enough, or problems related to illness or anatomical conditions such as swollen breasts or fat nipples. Peer support will assist first-time mothers to build their confidence in breastfeeding through experience sharing sessions. It will also help support mothers to overcome their breastfeeding challenges and problems.

Other intervention channels for providing education and support are using telephone contacts for discussion or counseling or the production of promotional materials such as leaflets, flip charts, DVDs, or workbooks. An experimental study in Brazil found that mothers who received educational interventions, enhanced by a flip chart with illustrations of breastfeeding themes, showed increased breastfeeding self-efficacy [36]. The use of checklists, pamphlets, and audiovisual materials on breastfeeding among recently delivered mothers in hospitals also improved the mothers’ self-efficacy for breastfeeding [37].

Mothers who delivered by Caesarean section in this study were more likely to have low breastfeeding self-efficacy. This finding was similar to other studies where women who had delivered by Caesarean section were less likely to breastfeed or to delay breastfeeding initiation [41, 42]. The problems related to lactogenesis due to abdominal surgery or stress response due to delivery complications, contributed to increased difficulty with or early cessation of breastfeeding [41, 43]. Consequently, interventions targeting mothers, both with planned or emergency cesarean section, are required. Support and counseling programs by health workers and lactation counselors, including postpartum home visits, might benefit these women.

Previous research reported the critical role of family support to help mothers to breastfeed their children [44]. Women who received praise from other family members had higher breastfeeding self-efficacy scores than those receiving praise from friends [45]. Thus, family members’ involvement, particularly husbands, in educational programs, for example, during prenatal classes, is vital for promoting optimal breastfeeding practices.

The odds of high self-efficacy with breastfeeding amongst women in this study increased as the mothers’ number of breastfeeding interventions increased. Many of BADUTA interventions aimed to strengthen the existing health system, which was also available to mothers in the comparison group. Consequently, in the endline survey, we found a high percentage of women from the comparison group also received breastfeeding interventions. These breastfeeding interventions heavily relied on awareness-raising and education strategies to improve the community’s knowledge and breastfeeding skills. The combined effect of different interventions might reflect a dose-response effect of increasing odds of higher breastfeeding self-efficacy with an increasing number of breastfeeding intervention services received by mothers [46, 47].

Efforts to ensure optimum quality of counseling on breastfeeding, educational interventions, and social supports received by mothers are essential to improve mothers’ breastfeeding practice. Therefore, programs should consider interventions to increase knowledge and skills of health workers, lactation counselors as well as community health workers (cadres), particularly concerning their counseling skills. Trained birth attendants, whose services are still widely used by the community in some settings for mother and childcare, could be trained by health workers to promote breastfeeding amongst pregnant and recently delivered mothers.

### The role of maternal occupation on breastfeeding self-efficacy

We found a low level of breastfeeding self-efficacy in women working outside their homes. Secondary analysis using nationally representative survey data for Indonesia found that a mother working outside her home was a barrier for optimal breastfeeding practices [48]. A strong reason for this could be the short duration of maternity leave in Indonesia. In the formal sector, only 1.5 months of maternity leave is given before and after delivery [49], while in the informal sector, this regulation is often not fully applied. Although the Republic of Indonesia’s Act stated that female workers still breastfeeding their children should be given appropriate opportunities to breastfeed even during working hours [49], supportive breastfeeding facilities are limited or even unavailable. Advocacy and the development of supportive policy and regulations to ensure the availability of lactation space and breastfeeding breaks in the workplace are crucial for women working outside their homes [47].

### Strengths and limitations

This study has several strengths. It has a large sample size, giving adequate power to analyze the role of different predictors on breastfeeding self-efficacy. It is the first study in Indonesia to examine self-efficacy with breastfeeding in mothers of children aged less than 6 months. Some limitations are worth noting when interpreting the results. We based our results on the mothers’ recall ability, and we did not validate the information provided by respondents. Another limitation was how mothers responded to breastfeeding self-efficacy questions was likely influenced by their current feeding practices, particularly amongst those who decided not to breastfeed their infants. There are other possible determinants of mothers’ self-efficacy with breastfeeding that we did not analyze because the variables were not available in the dataset, such as the mothers’ previous breastfeeding experience or the level of family support. To partially address previous breastfeeding experience, we examined the association of having had a previous live birth on the odds of low breastfeeding self-efficacy in mothers. We found no association between them.

## Conclusions

Overall, our study found maternal education, knowledge of breastfeeding, occupation, and delivery mode as predictors of mothers’ breastfeeding self-efficacy. Consequently, multipronged breastfeeding education, breastfeeding counseling, and supports are required to increase women’s self-efficacy with breastfeeding that will accelerate appropriate breastfeeding practices despite the ongoing improvements in breastfeeding in Indonesia. Program managers should consider the use of combined settings, individual and group counseling with multiple contact opportunities. Effective education strategies and support programs targeting not only mothers but also other family members are required.

Additionally, efforts to ensure the availability and access to breastfeeding counselors or peer counselors will help mothers to increase their breastfeeding confidence. Training of cadres and traditional birth attendants to provide necessary counseling on breastfeeding will help to provide continuous support to women. Furthermore, home visits, including for those who delivered with cesarean section, will ensure mothers have ongoing support and overcome challenges or barriers to breastfeeding. Interventions to increase the availability of supporting facilities in the workplace are also required to enhance optimum breastfeeding practices amongst women working outside their homes.

## Supplementary Information

**Additional file 1: Table S1.** Factors associated with low breastfeeding self-efficacy amongst mothers of children under six months old, including specific breastfeeding problems, The BADUTA Study in East Java, Indonesia, 2015–2016.

**Additional file 2: Table S2.** Factors associated with low breastfeeding self-efficacy amongst mothers of children under six months old including specific breastfeeding interventions, The BADUTA Study in East Java, Indonesia, 2015–2016.

## Data Availability

The datasets used and/or analyzed during the current study are available from the corresponding author on reasonable request.
