# Supplementary material for: Determinants of low breastfeeding self-efficacy amongst mothers of children aged less than six months: results from the BADUTA study in East Java, Indonesia
Source: Int Breastfeed J. 2021 Jan 19;16:12. doi: 10.1186/s13006-021-00357-5 (PMC7816511; doi:10.1186/s13006-021-00357-5)
Supplement: Supplementary file 1 — Additional file 1: Table S1. Factors associated with low breastfeeding self-efficacy amongst mothers of children under six months old, including specific breastfeeding problems, The BADUTA Study in East Java, Indonesia, 2015–2016. [file 13006_2021_357_MOESM1_ESM.docx]

**Table S1.** Factors associated with low breastfeeding self-efficacy amongst mothers of children under six months old, including specific breastfeeding problems, The BADUTA Study in East Java, Indonesia, 2015-2016

| **Variable** | **Univariate** | | | | |  | | **Multivariate^1^** | | | | | | | |
| --- | --- | --- | --- | --- | --- | --- | --- | --- | --- | --- | --- | --- | --- | --- | --- |
|  | **aOR** | **95% CI** | | | ***p*** | |  | | **aOR** | **95% CI** | | | ***p*** | |  |
| **Contextual and intervention characteristics** | |  |  | |  | |  | |  |  |  |  | |  |  |
| **Exposure to intervention** |  |  |  | |  | |  | |  |  |  |  | |  |  |
| Exposed to intervention^2^ | 1.00 |  |  | |  | |  | |  |  |  |  | |  |  |
| Not exposed to intervention^3^ | 1.44 | 1.13 | 1.83 | | *0.003* | |  | |  |  |  |  | |  |  |
| **Number of breastfeeding interventions mothers exposed to** | | | |  | |  | |  | |  |  |  | |  |  |
| Three or more interventions | 1.00 |  |  | |  | |  | | 1.00 |  |  |  | |  |  |
| Two interventions | 1.53 | 0.83, | 2.81 | | *0.175* | |  | | 1.45 | 0.71, | 2.95 | *0.302* | |  |  |
| One intervention | 2.07 | 1.16, | 3.69 | | *0.014* | |  | | 1.79 | 0.97, | 3.29 | *0.062* | |  |  |
| No interventions | 2.42 | 1.41, | 4.15 | | *0.001* | |  | | 1.82 | 1.05, | 3.13 | *0.032* | |  |  |
| **Period** |  |  |  | |  | |  | |  |  |  |  | |  |  |
| Baseline | 1.00 |  |  | |  | |  | | 1.00 |  |  |  | |  |  |
| Endline | 0.73 | 0.55, | 0.97 | | *0.032* | |  | | 0.89 | 0.62, | 1.27 | *0.516* | |  |  |
| **Household characteristics** |  |  |  | |  | |  | |  |  |  |  | |  |  |
| **Household wealth index** |  |  |  | |  | |  | |  |  |  |  | |  |  |
| Poorest | 1.00 |  |  | |  | |  | |  |  |  |  | |  |  |
| Poor | 0.99 | 0.70, | 1.39 | | *0.948* | |  | |  |  |  |  | |  |  |
| Middle | 1.23 | 0.86, | 1.75 | | *0.257* | |  | |  |  |  |  | |  |  |
| Rich | 1.35 | 0.93, | 1.95 | | *0.118* | |  | |  |  |  |  | |  |  |
| Richest | 0.83 | 0.52, | 1.32 | | *0.426* | |  | |  |  |  |  | |  |  |
| **Mother's characteristics** |  |  |  | |  | |  | |  |  |  |  | |  |  |
| **Maternal age** |  |  |  | |  | |  | |  |  |  |  | |  |  |
| <19 years | 1.00 |  |  | |  | |  | |  |  |  |  | |  |  |
| 20-34 years | 0.70 | 0.41, | 1.17 | | *0.172* | |  | |  |  |  |  | |  |  |
| 35+ years | 0.67 | 0.40, | 1.14 | | *0.138* | |  | |  |  |  |  | |  |  |
| **Maternal education** |  |  |  | |  | |  | |  |  |  |  | |  |  |
| University/Academy | 1.00 |  |  | |  | |  | | 1.00 |  |  |  | |  |  |
| Completed senior high school | 1.69 | 1.19, | 2.39 | | *0.003* | |  | | 2.05 | 1.36, | 3.08 | *0.001* | |  |  |
| Completed junior high school | 1.94 | 1.31, | 2.86 | | *0.001* | |  | | 2.36 | 1.48, | 3.78 | *<0.001* | |  |  |
| No school/incomplete primary/completed primary school | 1.73 | 1.12, | 2.67 | | *0.014* | |  | | 1.97 | 1.21, | 3.21 | *0.007* | |  |  |
| **Maternal occupation** |  |  |  | |  | |  | |  |  |  |  | |  |  |
| Housework | 1.00 |  |  | |  | |  | | 1.00 |  |  |  | |  |  |
| Working outside the house | 1.38 | 1.06, | 1.81 | | *0.018* | |  | | 1.68 | 1.22, | 2.31 | *0.002* | |  |  |
| **Number of children still alive** |  |  |  | |  | |  | |  |  |  |  | |  |  |
| 1 | 1.00 |  |  | |  | |  | |  |  |  |  | |  |  |
| 2 | 0.99 | 0.77, | 1.26 | | *0.904* | |  | |  |  |  |  | |  |  |
| 3 | 1.02 | 0.69, | 1.52 | | *0.920* | |  | |  |  |  |  | |  |  |
| 4+ | 1.07 | 0.55, | 2.07 | | *0.847* | |  | |  |  |  |  | |  |  |
| **Previous live birth** |  |  |  | |  | |  | |  |  |  |  | |  |  |
| None | 1.00 |  |  | |  | |  | |  |  |  |  | |  |  |
| Any | 1.01 | 0.82, | 1.26 | | *0.906* | |  | |  |  |  |  | |  |  |
| **Antenatal and delivery care** |  |  |  | |  | |  | |  |  |  |  | |  |  |
| **Minimum antenatal care visits**^4^ |  |  |  | |  | |  | |  |  |  |  | |  |  |
| Completed (4+ visits) | 1.00 |  |  | |  | |  | | 1.00 |  |  |  | |  |  |
| Incomplete (<4 visits) | 1.28 | 0.98, | 1.68 | | 0.*073* | |  | | 1.27 | 0.95, | 1.71 | *0.112* | |  |  |
| **Mode of delivery** |  |  |  | |  | |  | |  |  |  |  | |  |  |
| Normal | 1.00 |  |  | |  | |  | | 1.00 |  |  |  | |  |  |
| Caesarean | 1.19 | 0.96, | 1.47 | | *0.108* | |  | | 1.34 | 1.06, | 1.70 | *0.017* | |  |  |
| **Birth attendant** |  |  |  | |  | |  | |  |  |  |  | |  |  |
| General practitioner/OBGYN | 1.00 |  |  | |  | |  | |  |  |  |  | |  |  |
| Midwife/nurse | 0.95 | 0.78, | 1.165 | | *0.631* | |  | |  |  |  |  | |  |  |
| Traditional birth attendant/family/friend | 1.29 | 0.54, | 3.082 | | *0.572* | |  | |  |  |  |  | |  |  |
| **Child's characteristics** |  |  |  | |  | |  | |  |  |  |  | |  |  |
| **Sex of the child** |  |  |  | |  | |  | |  |  |  |  | |  |  |
| Male | 1.00 |  |  | |  | |  | |  |  |  |  | |  |  |
| Female | 1.03 | 0.82, | 1.30 | | *0.791* | |  | |  |  |  |  | |  |  |
| **Birth weight from monitoring card** |  |  |  | |  | |  | |  |  |  |  | |  |  |
| Larger than average | 1.00 |  |  | |  | |  | |  |  |  |  | |  |  |
| Average | 0.72 | 0.51, | 1.00 | | *0.053* | |  | |  |  |  |  | |  |  |
| Smaller than average | 0.72 | 0.44, | 1.17 | | *0.183* | |  | |  |  |  |  | |  |  |
| **Breastfeeding knowledge & experience** |  |  |  | |  | |  | |  |  |  |  | |  |  |
| **Ever received any breastfeeding advice** |  |  |  | |  | |  | |  |  |  |  | |  |  |
| Yes | 1.00 |  |  | |  | |  | | 1.00 |  |  |  | |  |  |
| No | 1.40 | 1.09, | 1.78 | | *0.007* | |  | | 1.43 | 1.10, | 1.86 | *0.009* | |  |  |
| **Knowledge about breastfeeding** |  |  |  | |  | |  | |  |  |  |  | |  |  |
| High level^5^ | 1.00 |  |  | |  | |  | | 1.00 |  |  |  | |  |  |
| Low level^6^ | 1.58 | 1.19, | 2.10 | | *0.002* | |  | | 1.35 | 1.01, | 1.81 | *0.045* | |  |  |
| **Problems with breastfeeding** |  |  |  | |  | |  | |  |  |  |  | |  |  |
| **Swollen breast** | |  |  | |  | |  | |  |  |  |  | |  |  |
| No | 1.00 |  |  | |  | |  | | 1.00 |  |  |  | |  |  |
| Yes | 2.35 | 1.03, | 5.37 | | *0.043* | |  | | 3.10 | 1.33, | 7.21 | *0.009* | |  |  |
| **Sore nipples** | |  |  | |  | |  | |  |  |  |  | |  |  |
| No | 1.00 |  |  | |  | |  | | 1.00 |  |  |  | |  |  |
| Yes | 0.78 | 0.53, | 1.16 | | *0.217* | |  | | 0.79 | 0.50, | 1.24 | *0.305* | |  |  |
| **Flat nipples** | |  |  | |  | |  | |  |  |  |  | |  |  |
| No | 1.00 |  |  | |  | |  | |  |  |  |  | |  |  |
| Yes | 2.87 | 1.42, | 5.81 | | *0.004* | |  | | 2.75 | 1.29, | 5.87 | *0.009* | |  |  |
| **Infants refused breastfeeds** | | | |  | |  | |  | |  |  |  | |  |  |
| No | 1.00 |  |  | |  | |  | | 1.00 |  |  |  | |  |  |
| Yes | 7.18 | 2.16, | 23.79 | | *0.001* | |  | | 5.45 | 1.64, | 18.13 | *0.006* | |  |  |
| **Perceived breastmilk is not enough** | | | | | |  | |  | |  |  |  | |  |  |
| No | 1.00 |  |  | |  | |  | | 1.00 |  |  |  | |  |  |
| Yes | 2.75 | 2.03, | 3.74 | | *<0.001* | |  | | 2.80 | 2.04, | 3.84 | *<0.001* | |  |  |

*Note:*

*^1^Multivariate logistic regression using the backward elimination method to select significant predictors of low breastfeeding self-efficacy. The variable for the minimum requirement of four antenatal care visits by trimester was selected a priori to be retained in the final model regardless of its significance level. ^2^" Exposed to intervention," refers to respondents living in the intervention sub-districts at the endline survey ^3^Not exposed to intervention referred to all respondents from the baseline survey and those living in the control sub-districts at the endline survey. ^4^Minimum Antenatal Care refers to the recommendation of at least four antenatal visits, i.e., once in trimester one to three, and twice in trimester three. ^5^High level of knowledge was mothers whose total knowledge score was greater than, or equal to the median knowledge score value. ^6^Low level of knowledge was mothers whose total knowledge score was less than the median knowledge score value.*
