# Supplementary material for: Determinants of low breastfeeding self-efficacy amongst mothers of children aged less than six months: results from the BADUTA study in East Java, Indonesia
Source: Int Breastfeed J. 2021 Jan 19;16:12. doi: 10.1186/s13006-021-00357-5 (PMC7816511; doi:10.1186/s13006-021-00357-5)
Supplement: Supplementary file 2 — Additional file 2: Table S2. Factors associated with low breastfeeding self-efficacy amongst mothers of children under six months old including specific breastfeeding interventions, The BADUTA Study in East Java, Indonesia, 2015–2016. [file 13006_2021_357_MOESM2_ESM.docx]

**Table S2:** Factors associated with low breastfeeding self-efficacy amongst mothers of children under six months old including specific breastfeeding interventions, The BADUTA Study in East Java, Indonesia, 2015-2016

| **Variable** | **Univariate** | | | |  | **Multivariate^1^** | | | | | |
| --- | --- | --- | --- | --- | --- | --- | --- | --- | --- | --- | --- |
|  | **aOR** | **95% CI** | | ***p*** |  | **aOR** | **95% CI** | | | ***p*** |  |
| **Contextual and intervention characteristics** | | | |  |  |  |  |  | |  |  |
| **Exposure to intervention** |  |  |  |  |  |  |  |  | |  |  |
| Exposed to intervention^2^ | 1.00 |  |  |  |  |  |  |  | |  |  |
| Not exposed to intervention^3^ | 1.44 | 1.13, | 1.83 | *0.003* |  |  |  |  | |  |  |
| **Period** |  |  |  |  |  |  |  |  | |  |  |
| Baseline | 1.00 |  |  |  |  | 1.00 |  |  | |  |  |
| Endline | 0.73 | 0.55, | 0.97 | *0.032* |  | 0.97 | 0.65, | 1.43 | | *0.867* |  |
| **Ever visited by village facilitator at home** | | |  |  |  |  |  |  | |  |  |
| No | 1.00 |  |  |  |  | 1.00 |  |  | |  |  |
| Yes | 0.43 | 0.17, | 1.12 | *0.084* |  | 0.97 | 0.34, | 2.75 | | *0.948* |  |
| **Ever attended "emo-demo"** |  |  |  |  |  |  |  |  | |  |  |
| No | 1.00 |  |  |  |  | 1.00 |  |  | |  |  |
| Yes | 0.59 | 0.35, | 1.00 | *0.049* |  | 1.17 | 0.61, | 2.23 | | *0.635* |  |
| **Ever received SMS Bunda messages** | | |  |  |  |  |  |  | |  |  |
| No | 1.00 |  |  |  |  | 1.00 |  |  | |  |  |
| Yes | 0.41 | 0.15, | 1.14 | *0.087* |  | 0.56 | 0.16, | 1.94 | | *0.360* |  |
| **Ever visited by cadre at home** |  |  |  |  |  |  |  |  | |  |  |
| No | 1.00 |  |  |  |  | 1.00 |  |  | |  |  |
| Yes | 0.32 | 0.12, | 0.84 | *0.022* |  | 0.28 | 0.09, | 0.85 | | *0.025* |  |
| **Ever attended pregnancy class** |  |  |  |  |  |  |  |  | |  |  |
| No | 1.00 |  |  |  |  | 1.00 |  |  | |  |  |
| Yes | 0.50 | 0.21, | 1.21 | *0.125* |  | 0.87 | 0.33, | 2.25 | | *0.767* |  |
| **Roomed in with baby after delivery** | | |  |  |  |  |  |  | |  |  |
| No | 1.00 |  |  |  |  | 1.00 |  |  | |  |  |
| Yes | 0.62 | 0.49, | 0.78 | *0.000* |  | 0.75 | 0.56, | 1.01 | | *0.063* |  |
| **Ever received counselling by a midwife** | | |  |  |  |  |  |  | |  |  |
| No | 1.00 |  |  |  |  | 1.00 |  |  | |  |  |
| Yes | 0.81 | 0.59, | 1.11 | *0.185* |  | 1.17 | 0.83, | 1.65 | | *0.371* |  |
| **Ever received counselling by a cadre** | |  |  |  |  |  |  |  | |  |  |
| No | 1.00 |  |  |  |  | 1.00 |  |  | |  |  |
| Yes | 0.45 | 0.22, | 0.95 | *0.035* |  | 0.74 | 0.33, | 1.65 | | *0.463* |  |
| **Ever seen "*Rumpi Sehat*" TV commercials** | | | |  |  |  |  |  | |  |  |
| No | 1.00 |  |  |  |  | 1.00 |  |  | |  |  |
| Yes | 0.57 | 0.38, | 0.84 | *0.005* |  | 0.72 | 0.42, | 1.22 | | *0.216* |  |
| **Household characteristics** |  |  |  |  |  |  |  | |  |  |  |
| **Household wealth index** |  |  |  |  |  |  |  | |  |  |  |
| Poorest | 1.00 |  |  |  |  |  |  | |  |  |  |
| Poor | 0.99 | 0.70, | 1.39 | *0.948* |  |  |  | |  |  |  |
| Middle | 1.23 | 0.86, | 1.75 | *0.257* |  |  |  | |  |  |  |
| Rich | 1.35 | 0.93, | 1.95 | *0.118* |  |  |  | |  |  |  |
| Richest | 0.83 | 0.52, | 1.32 | *0.426* |  |  |  | |  |  |  |
| **Mother's characteristics** |  |  |  |  |  |  |  | |  |  |  |
| **Maternal age** |  |  |  |  |  |  |  | |  |  |  |
| <19 years | 1.00 |  |  |  |  |  |  | |  |  |  |
| 20-34 years | 0.70 | 0.41, | 1.17 | *0.172* |  |  |  | |  |  |  |
| 35+ years | 0.67 | 0.40, | 1.14 | *0.138* |  |  |  | |  |  |  |
| **Maternal education** |  |  |  |  |  |  |  | |  |  |  |
| University/Academy | 1.00 |  |  |  |  | 1.00 |  | |  |  |  |
| Completed senior high school | 1.69 | 1.19, | 2.39 | *0.003* |  | 1.92 | 1.28, | | 2.87 | *0.002* |  |
| Completed junior high school | 1.94 | 1.31, | 2.86 | *0.001* |  | 2.17 | 1.39, | | 3.37 | *0.001* |  |
| No school/incomplete primary/completed primary school | 1.73 | 1.12, | 2.67 | *0.014* |  | 1.85 | 1.16, | | 2.97 | *0.011* |  |
| **Maternal occupation** |  |  |  |  |  |  |  | |  |  |  |
| Housework | 1.00 |  |  |  |  | 1.00 |  | |  |  |  |
| Working outside the house | 1.38 | 1.06, | 1.81 | *0.018* |  | 1.72 | 1.25, | | 2.36 | *0.001* |  |
| **Number of children still alive** |  |  |  |  |  |  |  | |  |  |  |
| 1 | 1.00 |  |  |  |  |  |  | |  |  |  |
| 2 | 0.99 | 0.77, | 1.26 | *0.904* |  |  |  | |  |  |  |
| 3 | 1.02 | 0.69, | 1.52 | *0.920* |  |  |  | |  |  |  |
| 4+ | 1.07 | 0.55, | 2.07 | *0.847* |  |  |  | |  |  |  |
| **Previous live birth** |  |  |  |  |  |  |  | |  |  |  |
| None | 1.00 |  |  |  |  |  |  | |  |  |  |
| Any | 1.01 | 0.82, | 1.26 | *0.906* |  |  |  | |  |  |  |
| **Antenatal and delivery care** |  |  |  |  |  |  |  | |  |  |  |
| **Minimum antenatal care visits**^4^ |  |  |  |  |  |  |  | |  |  |  |
| Completed (4+ visits) | 1.00 |  |  |  |  | 1.00 |  | |  |  |  |
| Incomplete (<4 visits) | 1.28 | 0.98, | 1.68 | *0.073* |  | 1.21 | 0.90, | | 1.64 | *0.209* |  |
| **Mode of delivery** |  |  |  |  |  |  |  | |  |  |  |
| Normal | 1.00 |  |  |  |  | 1.00 |  | |  |  |  |
| Caesarean | 1.19 | 0.96, | 1.47 | *0.108* |  | 1.37 | 1.08, | | 1.74 | *0.011* |  |
| **Birth attendant** |  |  |  |  |  |  |  | |  |  |  |
| General practitioner/OBGYN | 1.00 |  |  |  |  |  |  | |  |  |  |
| Midwife/nurse | 0.95 | 0.78, | 1.165 | *0.631* |  |  |  | |  |  |  |
| Traditional birth attendant/family/friend | 1.29 | 0.54, | 3.082 | *0.572* |  |  |  | |  |  |  |
| **Child's characteristics** |  |  |  |  |  |  |  | |  |  |  |
| **Sex of the child** |  |  |  |  |  |  |  | |  |  |  |
| Male | 1.00 |  |  |  |  |  |  | |  |  |  |
| Female | 1.03 | 0.82, | 1.30 | *0.791* |  |  |  | |  |  |  |
| **Birth weight from monitoring card** |  |  |  |  |  |  |  | |  |  |  |
| Larger than average | 1.00 |  |  |  |  |  |  | |  |  |  |
| Average | 0.72 | 0.51, | 1.00 | *0.053* |  |  |  | |  |  |  |
| Smaller than average | 0.72 | 0.44, | 1.17 | *0.183* |  |  |  | |  |  |  |
| **Breastfeeding knowledge & experience** | | | |  |  |  |  | |  |  |  |
| **Ever received any breastfeeding advice** | | |  |  |  |  |  | |  |  |  |
| Yes | 1.00 |  |  |  |  | 1.00 |  | |  |  |  |
| No | 1.40 | 1.09, | 1.78 | *0.007* |  | 1.47 | 1.11, | | 1.93 | *0.006* |  |
| **Knowledge about breastfeeding** | |  |  |  |  |  |  | |  |  |  |
| High level^5^ | 1.00 |  |  |  |  | 1.00 |  | |  |  |  |
| Low level^6^ | 1.58 | 1.19, | 2.10 | *0.002* |  | 1.39 | 1.04, | | 1.87 | *0.029* |  |
| **Problems with breastfeeding** |  |  |  |  |  |  |  | |  |  |  |
| None | 1.00 |  |  |  |  | 1.00 |  | |  |  |  |
| Not related to illness | 3.14 | 2.34, | 4.23 | *<0.001* |  | 3.40 | 2.54, | | 4.56 | *<0.001* |  |
| Related to illness/anatomical condition | 1.28 | 0.91, | 1.80 | *0.158* |  | 1.35 | 0.95, | | 1.93 | *0.098* |  |
| Both types of problems | 3.18 | 1.29, | 7.81 | *0.012* |  | 3.73 | 1.33, | | 10.43 | *0.012* |  |

*Note:*

*^1^Multivariate logistic regression using the backward elimination method to select significant predictors of low breastfeeding self-efficacy. The variable for the minimum requirement of four antenatal care visits by trimester was selected a priori to be retained in the final model regardless of its significance level. ^2^Exposed to intervention refers to respondents living in the intervention sub-districts at the endline survey ^3^Not exposed to intervention refers to all respondents from the baseline survey and those living in the control sub-districts at the endline survey. ^4^Minimum antenatal care refers to the recommendation of at least four antenatal visits, i.e., once in trimester one to three, and twice in trimester three. ^5^High level of knowledge was mothers whose total knowledge score was greater than, or equal to the median knowledge score value. ^6^Low level of knowledge was mothers whose total knowledge score was less than the median knowledge score value.*
